# *Ex-Vivo* Tissues Engineering Modeling for Reconstructive Surgery Using Human Adult Adipose Stem Cells and Polymeric Nanostructured Matrix

**DOI:** 10.3390/nano6040057

**Published:** 2016-03-31

**Authors:** Francesco Morena, Chiara Argentati, Eleonora Calzoni, Marino Cordellini, Carla Emiliani, Francesco D’Angelo, Sabata Martino

**Affiliations:** 1 Department of Chemistry, Biology and Biotechnologies, Biochemistry and Molecular Biology Unit, University of Perugia, Via del Giochetto, Perugia 06122, Italy; effemorena@gmail.com (F.M.); chiara.argentati89@gmail.com (C.A.); eleonoracalzoni@gmail.com (E.C.); carla.emiliani@unipg.it (C.E.); francesco.dangelo@angelantoni.it (F.D.); 2 Unità Operativa Chirurgia Plastica e Ricostruttiva, ASL 1 Umbria, Città di Castello 06012, Italy; marinocordellini@yahoo.it

**Keywords:** adult mesenchymal stem cells isolation and culture, stem cells and biomaterial interaction, INTEGRA^®^, regenerative medicine

## Abstract

The major challenge for stem cell translation regenerative medicine is the regeneration of damaged tissues by creating biological substitutes capable of recapitulating the missing function in the recipient host. Therefore, the current paradigm of tissue engineering strategies is the combination of a selected stem cell type, based on their capability to differentiate toward committed cell lineages, and a biomaterial, that, due to own characteristics (e.g., chemical, electric, mechanical property, nano-topography, and nanostructured molecular components), could serve as active scaffold to generate a bio-hybrid tissue/organ. Thus, effort has been made on the generation of *in vitro* tissue engineering modeling. Here, we present an *in vitro* model where human adipose stem cells isolated from lipoaspirate adipose tissue and breast adipose tissue, cultured on polymeric INTEGRA^®^ Meshed Bilayer Wound Matrix (selected based on conventional clinical applications) are evaluated for their potential application for reconstructive surgery toward bone and adipose tissue. We demonstrated that human adipose stem cells isolated from lipoaspirate and breast tissue have similar stemness properties and are suitable for tissue engineering applications. Finally, the overall results highlighted lipoaspirate adipose tissue as a good source for the generation of adult adipose stem cells.

## 1. Introduction

Regeneration of *bona fide* soft or hard tissues is critical for reconstruction of several damaged tissues (due to, e.g., breast cancer defects, soft tissue augmentation, facial defects, bone cancer, and osteo-degenerative diseases). Nowadays, the gold standard for soft or hard tissue reconstruction is based on autologous tissue graft, a medical practice still associated with donor-site morbidity and volume reduction [1,2,3,4]. Therefore, a growing research in the world aimed at developing more suitable strategies for tissue regeneration [5,6,7].

In this regard, the tissue engineering approaches could be a hold promise [5,6,7]. This biotechnology combines the own therapeutic potential of stem cells, to replace healthy differentiated cells within the host tissue/organ, and biomaterials, to recreate the three-dimensional (3D) architecture of the tissue providing signals to guide the stem cell differentiation (mechanotransduction mechanisms) [8,9,10,11,12,13,14,15,16,17,18,19,20].

Therefore, effort has currently been made on: (i) selection of a human adult stem cell type, easy to isolate from host donor tissues and simple to expand and differentiate; and (ii) design of scaffolds based on biomaterials that could mimic the tissue that has to be regenerated.

It has been demonstrated that a successful tissue engineering scaffold should exhibit proper physical and mechanical characteristics and provide an appropriate surface chemistry with nano- and micro-structures to facilitate cellular adhesion, proliferation, and differentiation [10,11,12,13].

In this work, we have focused on adult human adipose mesenchymal stem cells (hASCs). These stem cells during the last ten years have attracted highest interest since they may be easily isolated from human subcutaneous adipose tissue [21,22] and have been demonstrated to have great regenerative potential toward different tissues, including multi damaged tissues after prostatectomy [23]; plastic surgery [24]; heart regeneration [25]; bone [26]; cartilage [27]; and skeletal muscle [28].

Of note, due to the different anatomic abundance and location of adipose tissue hASCs are still under ongoing comparative study to establish if they have the same cell phenotype independent of the adipose tissue origin [21,29,30]. Thus, elucidating this issue could have a deep impact on stem cell replacement therapeutic approaches since it will allow the isolation of hASCs from the adipose tissue that requires less invasive surgery with low economic impact in public health.

With this aim, here we have isolated hASCs from lipoaspirate adipose tissue and breast adipose tissue and compared their phenotypic characteristic and tissue engineering potential for developing an *ex-vivo* model using as scaffold a commercial available INTEGRA^®^, selected based on the nano-matrix polymeric components of the substrate as for its conventional clinical application for reconstructive surgery.

## 2. Results and Discussion

In the present study, we addressed whether human adipose stem cells (hASCs) isolated from adipose lipoaspirate tissue and adipose breast tissue could have comparable tissue engineering applications. To this end, we isolated hASCs from healthy lipoaspirate adipose tissue (L-ASCs) and healthy breast adipose tissue (B-ASCs) and evaluated the mesenchymal stem cell properties; then, we cultured both L-ASCs and B-ASCs on INTEGRA^®^ Meshed Bilayer Wound Matrix nanostructured polymer to assess the generation of *ex-vivo* transplantable tissues (adipose and bone) modeling.

### 2.1. Isolation and Characterization of Adult hASCs from Lipoaspirate Adipose Tissue and Breast Adipose Tissue

**Figure 1:**
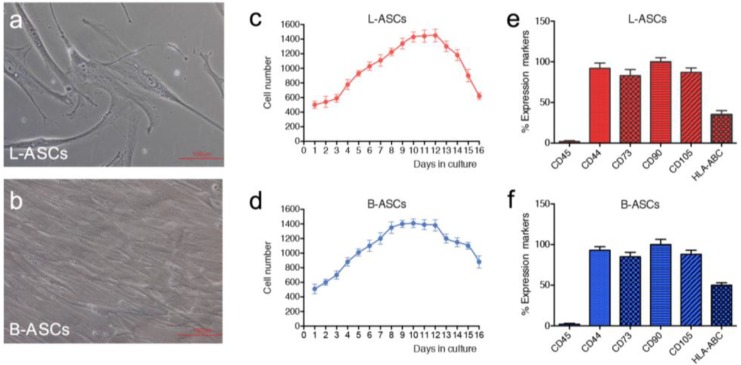
Isolation and characterization of hASCs (human adipose stem cells): L-ASCs (lipoaspirate-adipose stem cells) and B-ASCs (breast-adipose stem cells) were isolated from lipoaspirate adipose tissue and breast adipose tissue and cultured on TCP (tissue culture grade polystyrene) in growth medium. (**a**,**b**) Representative images of L-ASCs and B-ASCs showed the fibroblast-like morphology. Images were acquired with microscopy Eclipse-TE2000-S, Nikon (Nikon, Düsseldorf, Germany), using the F-ViewII FireWire^TM^ camera (Olympus Soft Imaging System, Münster, Germany); (**c**,**d**) L-ASCs and B-ASCs have similar growth curve. Cells were harvested and counted by TRYPAN BLUE reagent (Sigma-Aldrich, St Louis, MO, USA) every 24 h for 20 days using a hemocytometer; (**e**,**f**) Mesenchymal stem cell phenotype of L-ASCs and B-ASCs was analyzed using a FACScan flow cytometry (BD Biosciences, San Jose, CA, USA), and FlowJo software (Tree Star, Ashland, OR, USA) for data management.

hASCs were isolated from lipoaspirate and breast adipose tissue according to the procedure described in method section. In culture, both hASCs appeared as colonies with fibroblast-like morphology (Figure 1a,b). The growth rate, measured by performing the growth curve for 20 days, showed a doubling time of 94 h for L-ASCs and 91 h for B-ASCs (Figure 1c,d), thus indicating no significant differences in stem cell growth rate. Phenotypic analyses revealed comparable pattern expression of mesenchymal-specific surface antigens in L-ASCs and B-ASCs. Both stem cells were negative for CD45, according to their non-hematopoietic origin, and positive for mesenchymal surface markers: CD90, CD44, CD73 and CD105 (Figure 1e,f). Interestingly, both stem cells expressed human leukocyte antigen (HLA)-ABC (Figure 1e,f).

**Figure 2:**
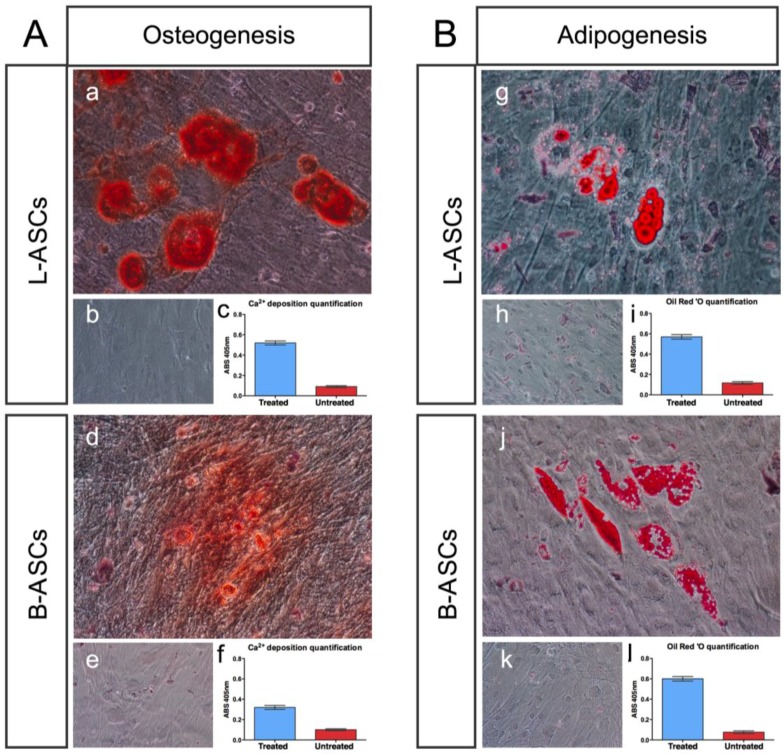
L-ASCs and B-ASCs have multipotential property. **Panel A**: L-ASCs (**a**–**c**) and B-ASCs (**d**–**f**) incubated in osteogenic medium differentiated towards osteogenic lineage as demonstrated by the Alizarin Red staining (**a**,**d**) and calcium quantification (**c**,**f**); and (**b**,**e**) untreated cells. **Panel B**: L-ASCs (**g,h,i**) and B-ASCs (**j**,**k**,**l**) incubated in adipogenic medium differentiated towards adipogenic lineage as demonstrated by the OIL Red staining (**g**,**j**) and lipid quantification (**i**,**l**); and (**h**,**k**) untreated cells. Representative images were acquired with microscopy Eclipse-TE2000-S, Nikon, using the F-ViewII FireWire™ camera (Soft Imaging System, Olympus).

hASCs multipotential property was evaluated by testing their osteogenic and adipogenic differentiation capability (Figure 2).

L-ASCs and B-ASCs, respectively, plated on tissue culture polystyrene (TCP) in osteogenic-inducing medium, differentiated toward osteocytes, as demonstrated by the accumulation of calcium (Alizarin Red staining) causing the presence of classical aggregates or nodules (Figure 2Aa; Figure 2Ad) that were absent in untreated cells (Figure 2Ab; Figure 2Ae). Of note, the visible difference in the rate of differentiation staining between L-ASCs and B-ASCs (Figure 2Aa; Figure 2Ad) was validated by the quantification data of the mineral deposition that was two-fold higher in L-ASCs compared to B-ASCs (Figure 2Ac; Figure 2Af), thus suggesting a highest differentiation response of hASCs isolated from lipoaspirate instead of breast adipose tissue.

After three weeks under adipogenic inducer medium, L-ASCs and B-ASCs differentiated toward adipogenic lineage, as indicated by the visible accumulation of lipid-rich droplets within cells and by lipid vacuoles that continued to develop over time, coalesced, and eventually also filling the cells (Figure 2Bg; Figure 2Bj), absent in untreated stem cells (Figure 2Bh; Figure 2Bk). We found no differences between the L-ASCs and B-ASCs differentiation rate toward adipocytic lineage on TCP as demonstrated by quantification of lipid deposition (Figure 2Bi; Figure 2Bl). These data were in agreement with the canonical function of these stem cells to generate/regenerate the adipose tissue *in vivo* tissue in physiological and in pathological condition [2,10,31].

**Figure 3:**
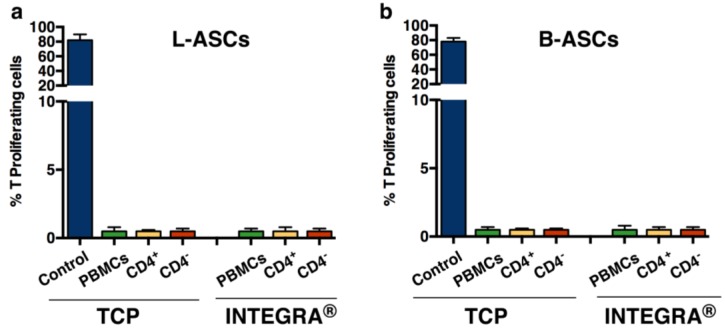
L-ASCs and B-ASC cultured on INTEGRA^®^ are hypo-immunogenic: L-ASCs (**a**) and B-ASC (**b**) cultured on INTEGRA^®^ and TCP are hypo-immunogenic as demonstrated by an MLR (Mixed Lymphocyte Reaction) assay. Control, indicating positive proliferation of CD4^+^T-cells after co-culture on immunogenic PBMCs (peripheral blood mononuclear cell).

Finally, we have tested the immunomodulatory property of hASCs, based on the number of studies showing the role of these and the other mesenchymal stem cell types, in immunomodulation [32,33]. We found that both L-ASCs and B-ASCs were hypo-immunogenic, since stem cells did not stimulate CD4^+^T proliferation as demonstrated by MLR (Mixed Lymphocyte Reaction) assay performed against autologous CD4^+^T cells (Figure 3a, TCP; Figure 3b, TCP).

The overall results indicated that adult hASCs isolated from lipoaspirate adipose tissue and breast adipose tissue have mesenchymal stem cell properties. However, it is likely that L-ASCs responded better to osteogenic differentiation rather than B-ASCs, thus suggesting that B-ASCs are more lineage committed compared to L-ASCs. Moreover, their hypo-immunogenic activity makes both stem cells suitable for safe *ex-vivo* tissue engineering applications.

### 2.2. INTEGRA^®^ Matrix is a Suitable Substrate for hASCs Culture

We explored the tissue engineering application of L-ASCs and B-ASCs by establishing an *ex-vivo* cell modeling where both stem cells were seeded on the commercially available polymeric substrate INTEGRA^®^ Meshed Bilayer Wound Matrix, already approved by FDA (Food and Drug Administration) for clinical practices. INTEGRA^®^ is a composite bilayer comprising cross-linked polymeric components that differ in their composition. Properly, the polymeric layers consist of a semi-permeable polysiloxane layer (to permit drainage and a flexible adherent surface) and a nano-matrix layer of bovine tendon collagen cross-linked with glycosaminoglycan (to guarantee cellular interaction, growth and proliferation even *in vivo* [34]. The nano-matrix layer was selected as surface for seeding stem cells due to the potential of collagen to establish direct contact with the focal adhesion proteins (e.g., vinculin) of cell membrane and to guide cytoskeleton architecture organization consequently.

**Figure 4:**
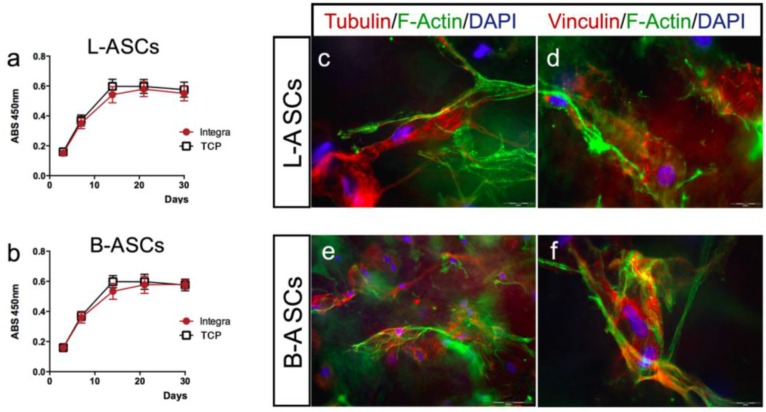
INTEGRA^®^ nanocomposite material is a suitable for culture of L-ASCs and B-ASCs. L-ASCs (**a**) and B-ASCs (**b**) cultured on INTEGRA^®^ have similar viability to stem cells cultured on TCP as demonstrated by 2,3-Bis-(2-Methoxy-4-Nitro-5-Sulfophenyl)-2H-Tetrazolium-5-Carboxanilide (XTT) assay measured at Absorbance (ABS) 450nm. L-ASCs (**c**,**d**) and B-ASCs (**e**,**f**) cultured on INTEGRA^®^ maintained the fibroblast-like morphology. Representative images of cytoskeleton fibers (F-Actin, Tubulin), focal adhesion spot (Vinculin) and nuclei (DAPI: diamidino-2-phenylindole) were acquired with fluorescence microscopy Eclipse-TE2000-S, Nikon, using the F-ViewII FireWire^TM^ camera (Soft Imaging System, Olympus).

First, we have evaluated the viability of L-ASCs and B-ASCs cultured on INTEGRA^®^, measuring the mitochondrial dehydrogenase activity at different time points (3, 7, 14, 21, and 30 days). We observed a comparable proliferation in stem cells growth on INTEGRA^®^ and TCP (Figure 4a,b). Moreover, no cell debris or signs of toxicity were observed in all cultures even after long-time in culture (data not shown).

Next, taking in mind the *in vivo* translational application of tissue engineering bio-hybrid and the potential immune system adverse effects causing rejection, we have evaluated the immune-modulatory activity of L-ASCs and B-ASCs cultured on INTEGRA^®^. According to previous observation on canonical TCP cultures, we confirmed the hypo-immunogenic activity of both stem cells (Figure 3a, INTEGRA^®^; Figure 3b, INTEGRA^®^). In fact, L-ASCs and B-ASCs cells did not stimulate CD4^+^T proliferation in an MLR assay (Figure 3a, INTEGRA^®^; Figure 3b, INTEGRA^®^). 

These data support the safe potential application of this bio-hybrid system for *in vivo* regenerative medicine approaches.

### 2.3. hASCs Cytoskeleton and Focal Adhesion on INTEGRA^®^ Matrix

It is a general concern that a successful tissue engineering therapy depends on the biomolecular interplay that is engaged between stem cells and the substrate in order to promote or guide the re-/generation of a specific differentiation lineage [8,9,10,35].

We have analyzed the interaction of stem cells with INTEGRA^®^ nano-matrix polymeric layer (collagen cross-linked with glycosaminoglycan component) by evaluating the cytoskeleton architecture (tubulin and F-actin) and focal adhesion spots (vinculin) by immunostaining (Figure 4c–f). Both hASCs seeded on the INTEGRA^®^ showed similar size and shape (Figure 4c–f). In particular, L-ASCs and B-ASCs seeded on INTEGRA^®^ have similar architecture of tubulin-positive fibers (microtubules) radiating out from the organizing center beside the nucleus with comparable orientation after 3, 7, and 21 days of culture (Figure 4b,d; representative images). Both hASCs cultured on INTEGRA^®^ showed F-actin–containing fibers arranged as straight, cable-like cords crossing the cytoplasm in multiple directions but preferentially oriented along the main cellular longitudinal axis (Figure 4c–f; representative images). Of note, these F-actin fibers frequently end with vinculin focal adhesion spots (Figure 4d,f; representative images), indicating canonical focal adhesion plaque organization [10,13] with the collagen component of polymeric nano-matrix layer (Figure 4c–f, representative images).

### 2.4. hASCs and INTEGRA^®^ for Generation of Osteogenic and Adipogenic Tissue

We have evaluated the capability of L-ASCs and B-ASCs seeded on INTEGRA^®^ to generate osteogenic and adipogenic tissues.

**Figure 5:**
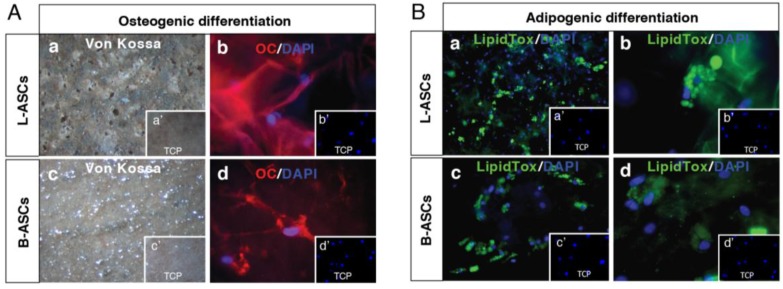
INTEGRA^®^ nanocomposite material is suitable for tissue-engineering L-ASC- and B-ASC-modeling. Panel A: L-ASCs (**a**,**b**) and B-ASCs (**c**,**d**) cultured on INTEGRA^®^ incubated in osteogenic medium differentiated towards osteogenic lineage as demonstrated by the Von Kossa staining (**a**,**c**) and Osteocalcin expression (**b**,**d**); and (**a’**–**d’**) untreated cells. Panel B: L-ASCs (**a**,**b**) and B-ASCs (**c**,**d**) cultured on INTEGRA^®^ incubated in adipogenic medium differentiated towards adipogenic lineage as demonstrated by the Lipid Tox fluorescent staining; and (**a’**–**d’**). untreated cells. Representative images were acquired with fluorescence microscopy Eclipse-TE2000-S, Nikon) using the F-ViewII FireWire™ camera (Soft Imaging System, Olympus).

L-ASCs and B-ASCs after three weeks of culture on INTEGRA^®^ matrix under osteogenic-specific culture conditions, differentiated towards osteogenic lineages as revealed by the appearance of deep blue-purple staining indicating a significant calcium deposition (Figure 5Aa; Figure 5Ac), and the expression of the osteogenic marker osteocalcin (Figure 5Ab; Figure 5Ad). We found no differences between the L-ASCs and B-ASCs rate of differentiation toward osteocytes. Of note, calcium accumulation occurred only by treating cultures with osteogenic differentiation medium (Figure 5Aa,b; Figure 5Ac,d) as shown by absence of differentiation signs in untreated cultures (Figure 5Aa’,b’; Figure 5Ac’,d’).

Adipogenic lineage differentiation was also obtained after treatment of L-ASCs and B-ASCs cultured INTEGRA^®^ under adipogenic inducer medium, as indicated by the visible accumulation of lipid-rich vacuoles within cells, highlighted by LipidTox staining (Figure 5Ba,c and Figure 5Bb,d, respectively). However, we found no differences between the L-ASCs and B-ASCs rate of differentiation toward adipocytes on INTEGRA^®^. Moreover, lipid accumulation occurred only by treating cultures with adipogenic differentiation medium (Figure 5Ba’,c’ and Figure 5Bb’,d’, respectively).

Altogether, these data demonstrated that were no major differences in adhesion, morphology and osteocytes/adipocytes differentiation rates of both primary L-ASCs and B-ASCs cultured on INTEGRA^®^ nano-matrix.

## 3. Materials and Methods

### 3.1. Isolation of Mesenchymal Stem Cells from Adipose Tissues

Lipoaspirate adipose tissue and breast adipose tissue were obtained from healthy donor patients that underwent plastic intervention, and that give written consent, according to ethical committee. Tissues were crushed and extensively washed in phosphate-buffered saline (PBS) containing 5% penicillin/streptomycin (P/S) (EuroClone, Pero, Italy), then the tissue fragments were incubated 40 min at 37 °C, 5% CO_2_, with 0.075% collagenase Type I prepared in PBS containing 2% P/S for tissue digestion and then neutralized by adding 5 mL of Dulbecco’s Modified Eagle Medium (DMEM) (EuroClone, Pero, Italy) containing 20% heat inactivated Fetal Bovine Serum (FBS) (EuroClone, Pero, Italy). The digest was centrifuged at 300× *g*, then pellet washed with PBS/2% P/S and centrifuged at 300× *g* for 5 min. Finally, the cell pellet was re-suspended in growth medium (DMEM supplemented with 10% FBS, 1% l-glutamine (EuroClone, Pero, Italy), 1% P/S) plated in tissue culture flasks (TCP) and incubated at 37 °C, 5% CO_2_. hASCs start to grow as adherent fibroblast-like cells. The medium was changed every three days.

### 3.2. Immunophenotypic Characterization

hASCs phenotype, either from lipoaspirate adipose tissue (L-ASCs) and breast adipose tissue (B-ASCs), was analyzed using flow cytometry. Cultured L-ASCs and B-ASCs were trypsinized, washed, re-suspended in PBS supplemented with 1% bovine serum albumin and incubated with FITC (Fluorescein isothiocyanate)- and PE (Phycoerythrin)-conjugated mono-clonal antibodies (mAbs) for 30 min at 48 °C in the dark. The mAbs used were anti-CD45, anti-CD44, anti-CD73, anti-CD90, anti-CD105, and Human Leukocyte Antigen (HLA)-ABC (BD Biosciences, San Jose, CA, USA). Stained cells were analyzed using a FACScan flow cytometry (BD Biosciences, San Jose, CA, USA), and data were analyzed using FlowJo software (Tree Star, Ashland, OR, USA) for data management. Cells were electronically gated according to light-scattering properties to discriminate cell debris. Isotype-matched antibodies (anti-IgG (anti-Immunoglobulin G)-FITC, anti-IgG-PE) were used as negative control, to exclude unspecific signals.

### 3.3. hASCs Growth Curve and Cell Viability

Stem cells growth curve was performed by seeding L-ASCs and B-ASCs on TCP at a starting concentration of 500 cells/mL in growth medium. Cells were harvested and counted by TRYPAN BLUE reagent (Sigma-Aldrich, St Louis, MO, USA) every 24 h for 30 days using a hemocytometer.

For the cell viability study, L-ASCs and B-ASCs were seeded on TCP at a starting concentration of 500 cells/mL in growth medium. At different times (3, 7, 14, and 27 days), cell viability was evaluated by assaying the mitochondrial dehydrogenase activity by incubating cell cultures with 2,3-Bis-(2-Methoxy-4-Nitro-5-Sulfophenyl)-2H-Tetrazolium-5-Carboxanilide (XTT) salt solution (Sigma) for 4 h at 37 °C according to the manufacturer’s recommendations. The absorbance was measured using a microtiter plate reader (GDV, Rome, Italy) at 450 nm with a reference wavelength at 650 nm.

### 3.4. Multipotential Properties of hASCs

Multipotential capability of hASCs was analyzed testing the osteogenic and adipogenic differentiation seeding on TCP at a density of 2000 cells/cm^2^. For the first 24 h, cells were cultured in growth medium, then, were incubated with specific selected differentiation medium.

*Osteogenic differentiation* [36] was achieved using hMSC differentiation basal medium supplemented with osteogenic SingleQuots (Lonza Walkersville, Inc., Walkersville, MD, USA): dexamethasone, *l*-glutamine, ascorbate, pen/strep, mesenchymal cell growth supplement (MCGS), and b-glycerophosphate. Untreated cultures were maintained in growth medium. All cultures were maintained for 21 days in a humidified incubator at 37 °C and 5% CO_2_, with medium changes every 3 days.

To accomplish *adipogenic differentiation* [36], three cycles of induction and maintenance medium (Lonza Walkersville, Inc., Walkersville, MD, USA) were performed. Each cycle consisted of feeding the hASCs for 3 days (37 °C, 5% CO_2_) with supplemented adipogenesis induction medium (containing: rh-insulin, l-glutamine, MCGS, dexamethasone, indomethacin, 3-isobuty-lmethylxanthine, penicillin/streptomycin) followed by 1 to 3 days of culture in supplemented adipogenic maintenance medium (basal medium supplemented with rh-insulin, *l*-glutamine, MCGS, penicillin/streptomycin). Untreated hASCs were cultured in growth medium. All cultures were maintained for 21 days in a humidified incubator at 37 °C and 5% CO_2_.

### 3.5. INTEGRA^®^ Matrix

INTEGRA^®^ Meshed Bilayer Wound Matrix is a polymeric bilayer consisting of a layer of semi-permeable polysiloxane (to permit drainage and a flexible adherent surface) and a layer of bovine tendon collagen cross-linked with glycosaminoglycan (to guarantee cellular invasion and capillary growth *in vivo*) [34] and approved by FDA for clinical practices.

### 3.6. hASCs-INTEGRA^®^ Matrix Culture and Interaction

L-ASCs and B-ASCs were seeded at 2000 cells/cm^2^ on INTEGRA^®^ Meshed Bilayer Wound Matrix square in growth medium. Cultures were monitored at different time points (3, 7, 14, 21 days) for stem cells viability (according the experimental plan and the method described above in Section 3.3) and stem cell–scaffold interaction.

### 3.7. Osteogenic and Adipogenic Differentiation of hASCs on INTEGRA^®^

hASCs were seeded on INTEGRA^®^ at a density of 2000 cells/cm^2^ in growth medium for the first 24 h, and set for osteogenic differentiation and adipogenic differentiation using the method described in Section 3.4 and analyzed as reported in Section 3.10, Section 3.12 and Section 3.13. Non-induced hASCs were cultured in growth medium. All cell-matrix cultures were maintained for 21 days in a humidified incubator at 37 °C and 5% CO_2_.

### 3.8. MLR Assay

In total, 15 × 10^3^ of each type of hASCs (L-ASCs and B-ASCs) were co-cultured for 5 days in 96-well plates, in triplicate, with 3 × 10^5^ allogenic, CD4^+^ T lymphocytes, CD4^-^ and total PBMCs.

Human peripheral blood mononuclear cells (PBMCs) were obtained by Ficoll density gradient centrifugation, as described in our previous work (Ficoll-Hypaque Pharmacia Biotech AB, Uppsala, Sweden) [37,38]. CD4^+^ cells were purified using immune-magnetic selection using a mini-MACS cell isolation kit (Milteny-Biotec GmbH, Bergisch Gladbach, Germany) according to the manufacturer’s instructions.

During the last 6 h of culture 20 µM of 5-bromo-2'-deoxyuridine (BrdU) (BrdU Flow Kits, BD Biosciences, San Jose, CA, USA) was added in each well and lymphocyte proliferation was assessed by fluorescence microscopy as BrdU incorporation by CD4^+^ lymphocytes [37,38]. Results are expressed as the percentage of proliferating (BrdU^+^) T lymphocytes.

### 3.9. Oil Red O Staining and Lipid Deposition Quantification

Oil Red O (ORO) (BioVision Inc., Milpitas, CA, USA) stain was used to assess adipogenic differentiation on TCP. Cells were fixed in 4% paraformaldeheyde for 10 min at room temperature, washed with PBS, rinsed with 60% isopropanol for 10 min at room temperature, washed in H_2_O distilled (H_2_Od), and stained with 500 mL of ORO working solution (ORO 0.3% in isopropanol mixed with H_2_Od (3:2)) for 20 min at room temperature. Stained cells were rinsed with H_2_Od, and nuclei were counterstained by the addition of Mayer’s Haematoxylin solution (Sigma) for 10 min. The cells were then washed in H_2_Od and observed under a microscope (Eclipse TS-100, Nikon, Düsseldorf, Germany) equipped with a digital sight Nikon camera.

For quantitative lipid deposition analysis, Oil Red was extracted from stained cells in 400 μL 100% isopropanol for 5 min under gently shaking. Colorimetric quantification of Oil Red extracted solution was measured using a microtiter plate reader (ELISA reader, GDV, Rome, Italy) at 492 nm wavelength. Unstained cells that underwent the same procedure were used as negative control. Protein quantification was used as the internal control [39].

### 3.10. Neutral Lipid Fluorescent Staining

To avoid no-specific signal, LipidTOX™ Green neutral lipid stain (Invitrogen, Molecular Probes, Grand Island, NY, USA) was used to assess the adipogenic differentiation on INTEGRA^®^ [40]. Cells were fixed in 4% paraformaldehyde for 10 min at room temperature. After initial washing with PBS and a final wash with H_2_Od, cells were stained with 250 L H_2_Od, cell LipidTOX™ Green neutral lipid stain solution for 20 min at room temperature. Stained samples were mounted and nuclei were counterstained with Vectashield with diamidino-2-phenylindole (DAPI) (Vector Laboratories Inc., Burlingame, CA, USA). Images were acquired using fluorescence microscopy (Eclipse-TE2000-S, Nikon, Düsseldorf, Germany) using the F-ViewII FireWire™ camera (Olympus Soft Imaging System, Münster, Germany).

### 3.11. Alizarin Red Staining and Quantification of Mineralization

Alizarin red staining (AR) was used to assess the osteogenic differentiation on TCP [11]. L-ASCs and B-ASCs cultured for 21 days in osteogenic differentiation medium were washed with PBS twice and fixed with 4% paraformaldehyde dissolved in PBS for 10 min at RT (room temperature). Cultures were washed in H_2_Od and then incubated with 500 μL of Alizarin red staining (Lonza Walkersville Inc, Walkersville, MD, USA) solution for 20 min at RT. Cells were washed twice with distilled water and photos were captured with a Canon digital camera (PowerShot G10, Canon, Tokyo, Japan) and bright field microscopy (Eclipse-TE2000-S, Nikon).

Quantitative analysis of Alizarin Red Staining was performed according to manufacturer’s instruction (Lonza Walkersville Inc, Walkersville, MD, USA). Briefly, Alizarin Red was extracted from stained cells in 400 μL 10% acetic acid and incubating for 30 min by shaking, heated to 85 °C for 10 min, transferred to ice for 5 min, and centrifuged at 20,000× *g* for 15 min. Colorimetric quantification of Alizarin Red extracted solution was measured using a microtiter plate reader (ELISA reader, GDV) at 405 nm and absorbance were referred to a Alizarin Red standards curve.

### 3.12. Von Kossa Staining

To avoid non-specific signal, Von Kossa calcium stain (ABCAM,^®^ Cambridge, UK) was used to assess the osteogenic differentiation on INTEGRA^®^ [36]. Briefly, cultures, were washed with PBS twice and fixed with 4% paraformaldehyde dissolved in PBS for 10 min at RT (room temperature), rinsed in several changes of distilled water and incubate sections with 1% silver nitrate solution in a clear glass coupling jar placed under ultraviolet light for 20 min. After several changes of distilled water, removing un-reacted silver with 5% sodium thiosulfate for 5 min, and further washing in distilled water, cover slips were mounted. Images were acquired with fluorescence microscopy (Eclipse-TE2000-S, Nikon) using the F-ViewII FireWire^TM^ camera (Soft Imaging System, Olympus). Images casting was made by Adobe Photoshop CS5 program (Adobe Systems, San Jose, CA, USA).

### 3.13. Immunofluorescences

Immunofluorescence experiments, cells were rinsed twice with PBS, fixed in 4% paraformaldehyde for 30 min and, after PBS washing, cells were permeabilized, blocked (PBS + 10% FBS, and 0.1% Triton X-100) for 1 h at RT (room temperature), and incubated with phalloidin (Alexa-fluor-488 phalloidin, Invitrogen, Grand Island, NY, USA), for 20 min then further incubated overnight at 4 °C with other primary antibodies: anti-α-tubulin, anti-osteocalcin (Santa-Cruz Biotechnology, Santa Cruz, CA, USA) and anti-vinculin (clone hVIN-1, Sigma-Aldrich, St Louis, MO, USA). Finally, after washing with PBS and staining with Alexa-Fluor-594 nm conjugated secondary antibodies (Invitrogen) for 1 h at RT, cover slips were mounted and nuclei were counterstained with Vectashield with DAPI (Vector Laboratories Inc., Burlingame, CA, USA).

Images were acquired with fluorescence microscopy (Eclipse-TE2000-S, Nikon) using the F-ViewII FireWire^TM^ camera (Olympus Soft Imaging System, Olympus, Münster, Germany). Image casting was made by Adobe Photoshop CS5 program.

## 4. Conclusions

To be successful, regenerative medicine applications have to recreate biological substitutes (e.g., stem cells and biomaterial) capable of mimicking the architecture and function of a selected tissue/organ. This requires the strict interaction of stem cells and biomaterial properties that in turn orchestrate molecular events recapitulating the canonical function of the target tissue [8,9,10].

In this regards, studies are ongoing to explore: (i) the effectiveness of a selected stem cell type, seeded on a biomaterial, to provide a functional tissue; (ii) how stem cells interact with biomaterials; (iii) the relevance of a selected biomaterial property to induce a specific stem cell response (mechanotransduction); and (iv) *ex-vivo* tissue engineering modeling to define the road map for the generation of biocompatible and safe bio-hybrid system capable to guarantee stem cell adhesion, proliferation and differentiation.

Here, we have generated a tissue engineering model using L-ASCs and B-ASCs and polymeric nanostructured INTEGRA^®^ matrix.

We have demonstrated that hASCs isolated from lipoaspirate adipose tissue and breast adipose tissue seeded on INTEGRA^®^ exhibited similar stemness properties. This result is in contrast with the hASCs cultured on TCP, where L-ASCs displayed a highest osteogenic differentiation rate compared to B-ASCs. We suggest that the similar multipotential capability of L-ASCs and B-ASCs on INTEGRA^®^ could also be an effect of the nano-structure matrix (collagen cross-linked with glycosaminoglycan) that enhance the stem cell characteristics even in B-ASCs.

The overall results indicated that combination of L-ASCs or B-ASCs with INTEGRA^®^ could be suitable for developing tissue engineering modeling. Of note, our results also highlight that lipoaspirate, which can be obtained easily and safely, is a good source for the generation of adult adipose stem cells.

## References

[1] Katzengold R., Topaz M., Gefen A. (2016). Tissue loads applied by a novel medical device for closing large wounds. J. Tissue Viability.

[2] Philips B.J., Marra K.G., Rubin J.P. (2014). Healing of grafted adipose tissue: Current clinical applications of adipose-derived stem cells for breast and face reconstruction. Wound Repair Regen..

[3] Narins R.S., Beer K. (2006). Liquid injectable silicone: A review of its history, immunology, technical considerations, complications, and potential. Plast. Reconstr. Surg..

[4] DeFatta R.J., Williams E.F. (2008). Fat transfer in conjunction with facial rejuvenation procedures. Facial Plast. Surg. Clin. N. Am..

[5] Yin X., Mead B.E., Safaee H., Langer R., Karp J.M., Levy O. (2016). Engineering Stem Cell Organoids. Cell Stem Cell.

[6] Mandrycky C., Wang Z., Kim K., Kim D.H. (2015). 3D bioprinting for engineering complex tissues. Biotechnol. Adv..

[7] Wobma H., Vunjak-Novakovic G. (2015). Tissue Engineering and Regenerative Medicine 2015: A Year in Review. Tissue Eng..

[8] Xu Y., Liu L., Laslett A.L., Esteban M.A. (2013). Cell reprogramming: Into the groove. Nat. Mater..

[9] Murphy W.L., McDevitt T.C., Engler A.J. (2014). Materials as stem cell regulators. Nat. Mater..

[10] Martino S., D’Angelo F., Armentano I., Kenny J.M., Orlacchio A. (2012). Stem cell-biomaterial interactions for regenerative medicine. Biotechnol. Adv..

[11] D’Angelo F., Armentano I., Cacciotti I., Tiribuzi R., Quattrocelli M., Del Gaudio C., Fortunati E., Saino E., Caraffa A., Cerulli G.G. (2012). Tuning multi/pluri-potent stem cell fate by electrospun poly (l-lactic acid)-calcium-deficient hydroxyapatite nanocomposite mats. Biomacromolecules.

[12] D’Angelo F., Tiribuzi R., Armentano I., Kenny J.M., Martino S., Orlacchio A. (2011). Mechanotransduction: Tuning stem cells fate. J. Funct. Biomater..

[13] D’angelo F., Armentano I., Mattioli S., Crispoltoni L., Tiribuzi R., Cerulli G.G., Palmerini C.A., Kenny J.M., Martino S., Orlacchio A. (2010). Micropatterned hydrogenated amorphous carbon guides mesenchymal stem cells towards neuronal differentiation. Eur. Cell Mater..

[14] Childs P.G., Boyle C.A., Pemberton G.D., Nikukar H., Curtis A.S., Henriquez F.L., Dalby M.J., Reid S. (2015). Use of nanoscale mechanical stimulation for control and manipulation of cell behaviour. Acta Biomater..

[15] Tsimbouri P.M. (2015). Adult Stem Cell Responses to Nanostimuli. J. Funct. Biomater..

[16] Santos L.J., Reis R.L., Gomes M.E. (2015). Harnessing magnetic-mechano actuation in regenerative medicine and tissue engineering. Trends Biotechnol..

[17] Chen W., Shao Y., Li X., Zhao G., Fu J. (2014). Nanotopographical surfaces for stem cell fate control: Engineering mechanobiology from the bottom. Nano Today.

[18] Hao J., Zhang Y., Jing D., Shen Y., Tang G., Huang S., Zhao Z. (2015). Mechanobiology of mesenchymal stem cells: Perspective into mechanical induction of MSC fate. Acta Biomater..

[19] Farouz Y., Chen Y., Terzic A., Menasché P. (2015). Concise Review: Growing Hearts in the Right Place: On the Design of Biomimetic Materials for Cardiac Stem Cell Differentiation. Stem Cells.

[20] McMurray R.J., Dalby M.J., Tsimbouri P.M. (2015). Using biomaterials to study stem cell mechanotransduction, growth and differentiation. J. Tissue Eng. Regen. Med..

[21] Zuk P.A., Zhu M., Ashjian P., De Ugarte D.A., Huang J.I., Mizuno H., Alfonso Z.C., Fraser J.K., Benhaim P., Hedrick M.H. (2002). Human adipose tissue is a source of multipotent stem cells. Mol. Biol. Cell.

[22] De Francesco F., Ricci G., D’Andrea F., Nicoletti G.F., Ferraro G.A. (2015). Human Adipose Stem Cells: From Bench to Bedside. Tissue Eng. Part B.

[23] Yiou R., Mahrouf-Yorgov M., Trébeau C., Zanaty M., Lecointe C., Souktani R., Zadigue P., Figeac F., Rodriguez A.M. (2015). Delivery of human mesenchymal adipose-derived stem cells restores multiple urological dysfunctions in a rat model mimicking radical prostatectomy damages through tissue-specific paracrine mechanisms. Stem Cells.

[24] Eun S.C. (2014). Stem Cell and Research in Plastic Surgery. J. Korean Med. Sci..

[25] Chen L., Qin F., Ge M., Shu Q., Xu J. (2014). Application of adipose-derived stem cells in heart disease. J. Cardiovasc. Transl. Res..

[26] Barba M., Cicione C., Bernardini C., Michetti F., Lattanzi W. (2013). Adipose-derived mesenchymal cells for bone regereneration: State of the art. BioMed Res. Int..

[27] Veronesi F., Maglio M., Tschon M., Aldini N.N., Fini M. (2014). Adipose-derived mesenchymal stem cells for cartilage tissue engineering: State-of-The-Art in *in vivo* studies. J. Biomed. Mater. Res. Part A.

[28] Peçanha R., Ribeiro M.B., Ferreira A.B.R., Moraes M.O., Zapata-Sudo G., Kasai-Brunswick T.H., Campos-de-Carvalho A.C., Goldenberg R.C., Werneck-de-Castro J.P.S. (2012). Adipose-derived stem-cell treatment of skeletal muscle injury. J. Bone Jt. Surg..

[29] Lin C.S., Xin Z.C., Deng C.H., Ning H., Lin G., Lue T.F. (2010). Defining adipose tissue-derived stem cells in tissue and in culture. Histol. Histopathol..

[30] Perbellini F., Gomes R.S., Vieira S., Buchanan D., Malandraki-Miller S., Bruyneel A.A., Fialho M.D.L.S., Ball V., Clarke K., Faggian G. (2015). Chronic High-Fat Feeding Affects the Mesenchymal Cell Population Expanded From Adipose Tissue but Not Cardiac Atria. Stem Cells Transl. Med..

[31] Mizuno H., Tobita M., Uysal A.C. (2012). Concise Review: Adipose-Derived Stem Cells as a Novel Tool for Future Regenerative Medicine. Stem Cells Cancer Stem Cells.

[32] Mattar P., Bieback K. (2015). Comparing the immunomodulatory properties of bone marrow, adipose tissue, and birth-associated tissue mesenchymal stromal cells. Front. Immunol..

[33] Montespan F., Deschaseaux F., Sensébé L., Carosella E.D., Rouas-Freiss N. (2014). Osteodifferentiated mesenchymal stem cells from bone marrow and adipose tissue express HLA-G and display immunomodulatory properties in HLA-mismatched settings: Implications in bone repair therapy. J. Immunol. Res..

[34] INTEGRA. http://www.integralife.com/index.aspx?redir=products#Oti.

[35] Martino S., Morena F., Barola C., Bicchi I., Emiliani C. (2014). Proteomics and Epigenetic Mechanism in Stem Cells. Curr. Proteom..

[36] Martino S., D’Angelo F., Armentano I., Tiribuzi R., Pennacchi M., Dottori M., Mattioli S., Caraffa A., Cerulli G.G., Kenny J.M. (2009). Hydrogenated amorphous carbon nanopatterned film designs drive human bone marrow mesenchymal stem cell cytoskeleton architecture. Tissue Eng. Part A.

[37] Martino S., Tiribuzi R., Ciraci E., Makrypidi G., D’Angelo F., Di Girolamo I., Gritti A., de Angelis G.M., Papaccio G., Sampaolesi M. (2011). Coordinated involvement of cathepsins S, D and cystatin C in the commitment of hematopoietic stem cells to dendritic cells. Int. J. Biochem. Cell Biol..

[38] Tiribuzi R., D’Angelo F., Berardi A.C., Martino S., Orlacchio A. (2012). Knock-down of HEXA and HEXB genes correlate with the absence of the immunostimulatory function of HSC-derived dendretic cells. Cell Biochem. Funct..

[39] Morena F., di Girolamo I., Emiliani C., Gritti A., Biffi A., Martino S. (2013). A new analytical bench assay for the determination of arylsulfatase a activity toward galactosyl-3-sulfate ceramide: Implication for metachromatic leukodystrophy diagnosis. Anal. Chem..

[40] Lizundia E., Sarasua J.R., D’Angelo F., Orlacchio A., Martino S., Kenny J.M., Armentano I. (2012). Biocompatible poly(L-lactide)/MWCNT nanocomposites: Morphological characterization, electrical properties, and stem cell interaction. Macromol. Biosci..

